# Breaking down the barriers: the gut microbiome, intestinal permeability and stress-related psychiatric disorders

**DOI:** 10.3389/fncel.2015.00392

**Published:** 2015-10-14

**Authors:** John R. Kelly, Paul J. Kennedy, John F. Cryan, Timothy G. Dinan, Gerard Clarke, Niall P. Hyland

**Affiliations:** ^1^Laboratory of Neurogastroenterology, APC Microbiome Institute, University College CorkCork, Ireland; ^2^Department of Psychiatry and Neurobehavioural Science, University College CorkCork, Ireland; ^3^Department of Anatomy and Neuroscience, University College CorkCork, Ireland; ^4^Department of Pharmacology and Therapeutics, University College CorkCork, Ireland

**Keywords:** gut microbiota, intestinal barrier, gut-brain axis, depression, probiotics, psychobiotics

## Abstract

The emerging links between our gut microbiome and the central nervous system (CNS) are regarded as a paradigm shift in neuroscience with possible implications for not only understanding the pathophysiology of stress-related psychiatric disorders, but also their treatment. Thus the gut microbiome and its influence on host barrier function is positioned to be a critical node within the brain-gut axis. Mounting preclinical evidence broadly suggests that the gut microbiota can modulate brain development, function and behavior by immune, endocrine and neural pathways of the brain-gut-microbiota axis. Detailed mechanistic insights explaining these specific interactions are currently underdeveloped. However, the concept that a “leaky gut” may facilitate communication between the microbiota and these key signaling pathways has gained traction. Deficits in intestinal permeability may underpin the chronic low-grade inflammation observed in disorders such as depression and the gut microbiome plays a critical role in regulating intestinal permeability. In this review we will discuss the possible role played by the gut microbiota in maintaining intestinal barrier function and the CNS consequences when it becomes disrupted. We will draw on both clinical and preclinical evidence to support this concept as well as the key features of the gut microbiota which are necessary for normal intestinal barrier function.

## The gut microbiome

It is increasingly recognized that the gut microbiome might influence the core symptoms of neuropsychiatric disorders and that it might be a tractable target for novel treatment options. The mutualistic co-evolution of microbes and the human body, composed of more than 90% microbial cells and 10 million microbial genes has led to the collective being described as a “superorganism” (Nicholson et al., 2005). The most heavily colonized area of the human body is the gut, with bacterial concentrations ranging from 10^1^–10^3^ cells per gram in the upper intestine to 10^11^–10^12^ per gram in the colon (O'Hara and Shanahan, 2006; Derrien and van Hylckama Vlieg, 2015). Although the functional significance of the microbiome has yet to be fully determined (Franzosa et al., 2014), it is clear that an interlinked symbiotic relationship exists between host and microbe (Ley et al., 2008). In terms of bacterial phyla found in the gut, *Firmicutes* (species such as *Lactobacillus, Clostridium, Enterococcus*) and *Bacteroidetes* (species such as *Bacteroides*) account for the majority (Dethlefsen et al., 2007), though the other phyla such as *Actinobacteria (Bifidobacteria), Proteobacteria (Escherichia coli), Fusobacteria, Verrucomicrobia*, and *Cyanobacteria* are also present (Eckburg et al., 2005; Qin et al., 2010). Differences exist between the microbiota composition between the gut lumen and the microbiota composition which lies in close proximity to the mucus layer. For instance, gram negative *Proteobacteria* and *Akkermansia muciniphila* (Verrucomicrobia), which use mucus as a carbon and nitrogen source, adhere and reside within the mucus layer (van Passel et al., 2011). This gradient can be differentially regulated by factors such as stress (Rozee et al., 1982; Swidsinski et al., 2005; Johansson et al., 2011, 2014). A more detailed examination of the gut microbiota composition is beyond the scope of this review and the interested reader is directed to a number of excellent recent reviews on the topic (Bron et al., 2012; Jeffery et al., 2012a; Lozupone et al., 2012; O'Toole, 2012). Studies using different, but complementary, gut-microbiota directed interventions (Germ Free (GF) rodents, antibiotics, probiotics, gastrointestinal (GI) infection studies, and fecal microbiota transplantation studies) have all suggested a number of possible brain-gut signaling pathways under the influence of the gut microbiota and capable of modulating brain and behavior (Rhee et al., 2009; Grenham et al., 2011; Cryan and Dinan, 2012, 2015; Collins and Bercik, 2013; Dinan and Cryan, 2013; McVey Neufeld et al., 2013; Mayer et al., 2014a). This review will discuss the complex relationship between the gut microbiome and intestinal barrier function and examine the possible implications for stress-related psychiatric disorders.

## Development, structure, and function of the intestinal epithelial barrier

The main function of the intestinal barrier is to regulate the absorption of nutrients, electrolytes and water from the lumen into the circulation and to prevent the entry of pathogenic microorganisms and toxic luminal substances (Farhadi et al., 2003). Furthermore, regulation of the exchange of molecules between the environment and the host through the intestinal barrier influences the equilibrium between tolerance and immunity to self and non-self-antigens (Fasano and Shea-Donohue, 2005; Fasano, 2011). From a structural perspective these functions are preserved by a number of features including a mucus layer and a monolayer of epithelial cells interconnected by tight junctions (Madara, 1998). The mucus layer containing secretory immunoglobulin (Ig) A and antimicrobial peptides covers the epithelial cell lining which functions to facilitate GI transport, and as a protective layer against bacterial invasion. The colonic mucus layer is composed of two layers, an outer and inner layer composed of gel forming highly glycosylated proteins termed mucins. These are produced and maintained by goblet cells which renew the inner mucus layer approximately every hour (Johansson et al., 2011). These dynamic processes are subject to extensive and continuous interplay with the gut microbiota, disruption of which may have implications for the sustenance of key barrier functions (Yu et al., 2012; Bischoff et al., 2014).

Tight junctions on the other hand are complex protein structures that consist of transmembrane proteins such as claudin, occludin, and tricullulin (Dörfel and Huber, 2012). These transmembrane proteins connect with the opposing plasma membrane, thereby forming a mechanical link between epithelial cells and establishing a barrier to paracellular diffusion of fluid and solutes (Ivanov et al., 2010). The structure of the intestinal barrier is formed by the end of the first trimester (Montgomery et al., 1999). Epithelial cells with microvilli, goblet and enteroendocrine cells, appear by week eight of gestation and tight junctions are detected from week ten (Louis and Lin, 2009). Functional development of the intestinal barrier continues in the post natal period and is influenced by both feeding mode and diet (Cummins and Thompson, 2002; Verhasselt, 2010). Disruptions in this process, as exemplified by the underdeveloped intestinal barrier of the premature infant, can predispose to immune disorders (Groschwitz and Hogan, 2009). Indeed, there is an overlapping developmental course of the gut microbiota and intestinal barrier. The gut microbiota in the initial days of life is unstable and not particularly diverse in its make-up. By age three, however, the microbiota composition resembles that of an adult-like profile (Voreades et al., 2014). Several other factors may also influence the trajectory of microbiota development including gestational age (Barrett et al., 2013), mode of delivery (Dominguez-Bello et al., 2010), type of feeding (Penders et al., 2006), antibiotic use (Persaud et al., 2014), and exposure to family members and pets (Fujimura et al., 2010; Marques et al., 2010). The intestinal barrier acts as a shield which can be modified by the gut microbiota (Tlaskalová-Hogenova et al., 2011; Jakobsson et al., 2015) or its metabolites (Elamin et al., 2013). The mechanisms underlying the regulation of the epithelial barrier are complex. Recent evidence also suggests a novel role for non-coding RNAs, such as microRNAs, as important intermediaries in the interactions between host epithelial cells, immune cells and the gut microbiota (Cichon et al., 2014; Runtsch et al., 2014). Alterations in the gut microbiota have been associated with concomitant gut barrier dysfunction in both intestinal (Camilleri et al., 2012; Bonfrate et al., 2013; Scaldaferri et al., 2013) and extra-intestinal disorders (Vaarala et al., 2008). However, the role of the gut microbiota in disrupting the intestinal barrier in stress-related neuropsychiatric conditions, such as depression, has not been fully investigated.

## Microbiome and the blood brain barrier

Structural similarities exist between the intestinal, placental and blood brain barriers (BBB; Doran et al., 2013). The BBB is a complex neurovascular unit (Bauer et al., 2014) consisting of central nervous system (CNS) endothelial cells which separate the lumen of blood vessels from the CNS parenchyma. Tight junctions, astrocytes and pericytes seal the capillary endothelial cells of the BBB (Daneman and Rescigno, 2009). The tight junctions transmembrane proteins claudins, tricellulin, and occludin restrict paracellular diffusion of water-soluble substances from blood to the brain (Hawkins and Davis, 2005). Recent preclinical evidence from GF mice suggests that the microbiota can modulate the BBB. Exposure of GF adult mice to the fecal microbiota from pathogen-free donors decreased BBB permeability and increased the expression of tight junction proteins (Braniste et al., 2014). Moreover, monocolonization of the intestine of GF adult mice with short chain fatty acid (SCFA)-producing bacterial strains normalized BBB permeability whilst sodium butyrate was associated with increased expression of occludin in the frontal cortex and hippocampus (Braniste et al., 2014). This study strengthens the hypothesis that the BBB may also be vulnerable to changes in the gut microbiota.

## Brain-gut-microbiota axis

Bidirectional signaling between the gut and the brain is regulated at neural, endocrine, and immune levels. These pathways are under the influence of the gut microbiota and together, they comprise the brain-gut-microbiota axis (Grenham et al., 2011). A cardinal function of the gut microbiota is the development and maintenance of the intestinal barrier across the lifespan (Ohland and Macnaughton, 2010; Swanson et al., 2011; Shifrin et al., 2012). It is plausible that subtle alterations in microbiota acquisition or maintenance in early life may act as a vulnerability factor, impacting on (neuro)endocrine and (neuro)immune signaling pathways of the brain-gut-microbiota axis, disruption of which may subsequently predispose to stress-related disorders in adulthood (Borre et al., 2014). Notably, animals devoid of a microbiota exhibit reduced levels of anxiety but an exaggerated neuroendocrine response to stress (Sudo et al., 2004). The most pronounced impacts of the microbiota may occur early in life during critical neurodevelopmental phases (Borre et al., 2014). It is evident that the gut microbiota is required for the normal development of the hypothalamic pituitary adrenal (HPA) axis and that there is a certain period in early life when colonization must occur to ensure normal development of this critical stress signaling pathway (Sudo et al., 2004; Moloney et al., 2014). Indeed, this concept is an extension of the hygiene hypothesis first proposed in the late 1980's (Strachan, 1989) and more recently reconceptualised as the “old friends hypothesis” (Rook et al., 2003). This proposes that encountering less microbial biodiversity may contribute to the increase in chronic inflammatory disorders including subtypes of depression (Klerman and Weissman, 1989; Weissman, 1992; Guarner et al., 2006; Rook and Lowry, 2008; Hidaka, 2012; Rook et al., 2013, 2014; Williamson et al., 2015).

The interaction between the immune system, the gut microbiota and the intestinal barrier may be of particular importance to health at the other extreme of life, aging. Aging is characterized by chronic low-grade inflammation (termed “inflammaging”) as evidenced by increased circulating levels of Tumor necrosis factor-alpha (TNF-α), interleukin (IL)-6 and C-reactive protein (CRP); inflammatory molecules known to affect mood and cognition (Frasca and Blomberg, 2015). The fact that the gut microbiota are key regulators of immune function and inflammatory responses, it is likely that a change in the composition of the gut microbiota during aging plays a role in the gradual activation of the immune system and consequently inflammaging (Prenderville et al., 2015), possibly via an impact on intestinal permeability. Indeed, the ELDERMET consortium demonstrated that the elderly have a distinct microbiota profile, characterized by greater inter-individual variation compared to younger adults (Claesson et al., 2011). Of note, differences in microbiota composition were more pronounced between frail elderly subjects and healthy elderly subjects. Moreover, certain gut microbiota signatures were linked to measures of frailty, co-morbidity, nutritional status, and markers of inflammation (Claesson et al., 2012).

## Stress, the gut microbiota and barrier function

Stress can impact on the developmental trajectory of the intestinal barrier (Smith et al., 2010; Lennon et al., 2013) and has been associated with an increase in gut permeability (Söderholm et al., 2002). Indeed, the effects of stress on intestinal permeability are complex and likely involve both the gut and the brain. Corticotrophin releasing factor (CRF) and its receptors (CRFR1 and CRFR2), play a key role in stress-induced gut permeability dysfunction (Overman et al., 2012; Rodiño-Janeiro et al., 2015; Taché and Million, 2015). In response to an acute stressor, colonic paracellular permeability increases and has been associated with the development of visceral hypersensitivity (Ait-Belgnaoui et al., 2005). In a mouse model of chronic depression, elevated central CRH expression occurred concomitantly with changes in the gut microbiota (Park et al., 2013). Early life stress has also been demonstrated to enhance plasma corticosterone in rat pups and is associated with an increase in intestinal permeability and bacterial translocation to the liver and spleen. This effect appeared to predominate in the colon (Moussaoui et al., 2014). Of note, stress-induced changes in the HPA axis and autonomic nervous system display sensitivity to probiotic intervention (*Lactobacillus helveticus* R0052 and *Bifidobacterium longum* R0175); (Ait-Belgnaoui et al., 2014). Moreover, these probiotics also restored colonic tight junction integrity in stressed mice (Ait-Belgnaoui et al., 2014). Probiotics have also been demonstrated to influence bacterial adhesion and translocation to mesenteric lymph nodes in response to stress (Zareie et al., 2006). *Lactobacillus farciminis* in particular not only suppresses stress-induced changes in permeability, HPA axis activity, endotoxaemia, and neuroinflammation (Ait-Belgnaoui et al., 2012), but also beneficially influences the mucus barrier (Da Silva et al., 2014). Human studies further confirm that acute-stress paradigms can affect intestinal permeability. In a public speaking based stressor, small intestinal permeability was significantly increased, however, this was only observed in those subjects who also responded with a significant elevation of cortisol (Vanuytsel et al., 2014). In a different acute stress model using a cold pain stressor, albumin permeability increased, though in females only (Alonso et al., 2012).

Stressful early-life events are strongly associated with the development of depression later in life (Heim et al., 2000). The interaction between stress, the HPA axis and the immune system is well established (Baes et al., 2014; Hueston and Deak, 2014). In recent years it has emerged that the gut microbiota mediates this interaction (De Palma et al., 2014b). Early-life maternal separation, for example, results in a significant decrease in fecal *Lactobacillus* numbers three days post-separation and this correlated with stress-related behaviors (Bailey and Coe, 1999). In a mouse model of social disruption, stress-induced alterations in the microbiota were accompanied by changes in cytokine and chemokine levels (Bailey et al., 2011). Several other studies have verified that stress can remodel the gut microbiota composition (Wang and Wu, 2005; O'Mahony et al., 2009; Galley et al., 2014a,b; De Palma et al., 2015). This is also relevant prenatally, as infants of mothers with high self-reported stress and high salivary cortisol concentrations during pregnancy had a significantly higher relative abundance of *Proteobacteria* and lower relative abundances of lactic acid bacteria (*Lactobacillus, Lactoccus, Aerococcus*) and *Bifidobacteria*. However, it is currently unclear whether this effect was mediated via maternal microbial transmission or through microbiota-independent events. Nonetheless, those infants with altered microbiota composition exhibited a higher level of infant GI symptoms and allergic reactions, highlighting the functional consequences of aberrant colonization patterns in early life (Zijlmans et al., 2015). Recent preclinical evidence showed that maternal stress altered the vaginal microbiota, decreasing *Lactobacillus*, with implications for the metabolic profile and neurodevelopment in the offspring (Jašarevic et al., 2015a,b).

**Figure 1:**
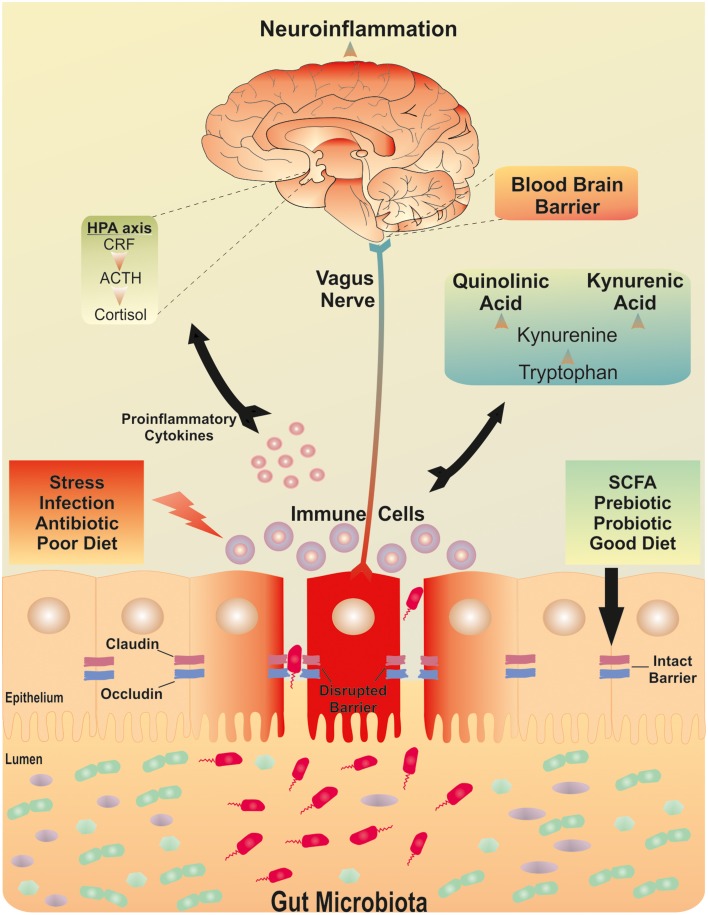
**The brain-gut-microbiota axis**. Postulated signaling pathways between the gut microbiota, the intestinal barrier and the brain. A dysfunctional intestinal barrier or “leaky gut” could permit a microbiota-driven proinflammatory state with implications for neuroinflammation.

A dysfunctional intestinal barrier could permit a microbiota driven proinflammatory state with implications for the brain (see Figure 1). The sequence of this process is not yet clear. An increase in gut permeability could precede mucosal inflammation to induce the inflammatory response and thus culminate in a feed-forward cycle between inflammatory responses and barrier dysfunction. This could subsequently maintain and exacerbate the low grade inflammatory response. Alternatively, systemic inflammation could increase intestinal barrier permeability and thus allow translocation of commensal bacteria with further implications for systemic inflammation. Indeed, the source of the low grade inflammation which has been reported in depression has not been isolated to a particular source. Irrespective of the sequence, both processes could engage the gut microbiota. Higher IgA- and IgM-mediated immune responses directed against lipopolysaccharides (LPS) of certain commensal gram negative gut bacteria have been shown in depressed patients (Maes et al., 2008, 2012). The implication being that the presence of such responses may have occurred subsequent to disruption of the intestinal barrier. Moreover, bacterial DNA has been detected in whole serum from depressed patients who also displayed increased Toll-like receptor (TLR)-4 expression on peripheral mononuclear blood cells compared to healthy controls (Kéri et al., 2014). To date, two published cross sectional studies have investigated the gut microbiota composition in depression. The first found an increase in *Bacteroidales* and a decrease in *Lachnospiraceae* compared to controls. However, there were no significant differences in species richness, α-diversity, or operational taxonomic units (Naseribafrouei et al., 2014). In the second study increased levels of *Enterobacteriaceae* and *Alistipes* were observed. Moreover, *Fecalibacterium* levels were reduced in the depressed group and negatively correlated with severity of depressive symptoms (Jiang et al., 2015). In the context of alcohol abuse, a relationship between the microbiota, barrier function and comorbid depression has recently been reported (Leclercq et al., 2014a). Microbiota-derived LPS and peptidoglycans (PGN) were demonstrated to cross the gut barrier and activate their respective receptors, TLR4 and TLR2 in peripheral blood mononuclear cells. Although chronic alcohol consumption inhibited the NF-κB pathway, it activated protein kinase/activator protein 1 pathway and IL-8 and IL-1B. In contrast, short term alcohol withdrawal was associated with the recovery of TLR4 receptors. The same group also demonstrated that increased intestinal permeability occurred in a sub group of alcohol-dependent subjects which were associated with higher depression and anxiety scores as well as an altered gut microbiota profile (Leclercq et al., 2014b).

## Irritable bowel syndrome

Irritable bowel syndrome (IBS) is a stress-related functional brain-gut-microbiota axis disorder associated with an altered gut microbiota profile (Carroll et al., 2011; Jeffery et al., 2012c; Collins, 2014; De Palma et al., 2014a; Simrén, 2014; Soares, 2014; Rajilic-Stojanovic et al., 2015) and increased intestinal permeability (Dunlop et al., 2006; Rao et al., 2011; Camilleri et al., 2012). Moreover, a significant proportion of IBS patients also suffer from depressive and anxiety symptoms (Singh et al., 2012; Lucas et al., 2014); furthermore, these psychiatric symptoms increase with the greater frequency and severity of GI symptoms (Pinto-Sanchez et al., 2015). In addition, alterations in brain circuits involved in attention, emotion, pain (Labus et al., 2009; Blankstein et al., 2010; Tillisch et al., 2013) together with deficits in hippocampal-mediated visuospatial memory (Kennedy et al., 2012, 2014) have been noted in the disorder. In particular, an altered *Firmicutes: Bacteroidetes* ratio has been linked to IBS and an association between *Firmicutes, Proteobacteria* and IBS symptom scores has been demonstrated (Rajilic-Stojanovic et al., 2011). A recent meta-analysis of clinical studies to identify and assess the various diagnostic tests indicated that a combination of intestinal permeability, Rome I criteria and fecal calprotectin (see Table 1) provided the highest positive likelihood ratio for predicting IBS (Sood et al., 2015). Although not captured in this meta-analysis, and not part of routine clinical practice, the addition of gut microbiota profiling may deliver further diagnostic accuracy (Casen et al., 2015). For example, IBS subtypes have been stratified according to their microbiota profiles, specifically those with an increased *Firmicutes: Bacteroidetes* ratio (Jeffery et al., 2012b). Furthermore, depression was the most robust clinical discriminator between a high *Firmicutes: Bacteroidetes* ratio in IBS patients relative to IBS patients with a healthy-like microbiota signature (Jeffery et al., 2012b). In addition, the order *Actinomycetales* and the family *Actinomycetaceae* were inversely associated with clinically significant depression (Jeffery et al., 2012b).

**Table 1:** **Markers of intestinal permeability**.

**Permeability test**	**Sample**	**Measures**	**Clinical/Preclinical**	**Representative citation**
**CHALLENGE TESTS**				
Lactulose/Mannitol	Urine	Small intestine permeability	Clinical and preclinical	Vanuytsel et al., 2014
Lactulose/L-rhamnose	Urine	Small intestine permeability	Clinical and preclinical	Keszthelyi et al., 2014
Sucrose	Urine	Gastric permeability	Clinical and preclinical	Mujagic et al., 2014
Sucralose	Urine	Colonic permeability	Clinical and preclinical	Anderson et al., 2004
Polyethylene glycols	Urine	Entire intestine permeability	Clinical and preclinical	Rao et al., 2011
^51^Cr-EDTA	Urine	Entire intestine permeability	Clinical and preclinical	Grootjans et al., 2010
**CIRCULATING MARKERS**				
Zonulin	Plasma	Small intestine epithelial cell damage	Clinical and preclinical	Fasano, 2011
Intestinal fatty acid binding protein (I-FABP)	Plasma	Small intestine permeability	Clinical and preclinical	Derikx et al., 2009
Citrulline	Plasma	Small intestine epithelial cell damage	Clinical and preclinical	Crenn et al., 2000
αGlutathione S-transferase (αGST)	Plasma	Epithelial cell damage	Clinical and preclinical	McMonagle et al., 2006
Claudin-3	Urine	Epithelial cell damage	Clinical and preclinical	Patel et al., 2012
Lipopolysacharide Binding protein (LBP)	Plasma	Indirect evidence of permeability deficit	Clinical and preclinical	Pasternak et al., 2010
Endotoxin core antibodies (EndoCAb)	Plasma	Entire intestine permeability	Clinical and preclinical	Ammori et al., 2003
D-Lactate	Plasma	Entire intestine permeability	Clinical and preclinical	Poeze et al., 1998
Fluorescein isothiocyanate—dextran (FITC-Dextran 4)	Plasma	Entire intestine permeability	Preclinical	Moussaoui et al., 2014
**FECAL MARKERS**				
Calprotectin	Feces	Nonspecific marker of gut inflammation	Clinical and preclinical	de Magistris et al., 2010
Zonulin	Feces	Marker of intestinal permeability	Clinical	Lamprecht et al., 2012
***EX VIVO***				
Ussing chamber	*Ex vivo* biopsies	Tranepithelial electrical resistance and macromolecule flux	Clinical and preclinical	Piche et al., 2009

Although the microbiota varies along the length of the gastrointestinal tract, the majority of studies use fecal microbiota sampling as a representative of global changes, however site specific changes may influence the ensuing immune consequences. For example mucosal jejunal tissue from diarrhea-predominant IBS patients is also associated with increased activation of mucosal B lymphocytes, plasma cells and mucosal IgG production (Vicario et al., 2015). Of note in this study, humoral activity markers positively correlated with depressive symptoms (Vicario et al., 2015). Further studies need to be conducted to disentangle the contributing role of an exaggerated or aberrant immune response, changes in intestinal permeability and psychiatric co-morbidities in IBS. However, individuals with pre-existing psychological disorders are known to be at an increased risk of developing post-infectious IBS (PI-IBS) in particular (Thabane and Marshall, 2009). Variations in several genes associated with bacterial recognition, the inflammatory response and epithelial integrity including TLR9, IL-6 and cadherin 1 genes have been identified as risk factors for the development of PI-IBS (Craig and Quigley, 2010; Villani et al., 2010). A longitudinal study which examined the rate of IBS development following an accidental outbreak of *E. coli* 0157:H7 into a town's (Walkerton) water supply identified an increased rate of IBS two years after the outbreak (Marshall, 2009). A subsequent study further identified a modest increase in intestinal permeability amongst this IBS cohort (Marshall et al., 2004); an association between this outbreak and depression was also identified (Garg et al., 2006). However, whether there is an association between a deficient barrier and the onset of depressive-like symptoms amongst this cohort has yet to be determined. Similarly, an outbreak of Shiga toxin-producing *E. coli* O104 in Germany increased self-reported depressive and anxiety symptoms measured six months after the infection (Löwe et al., 2014). However, intestinal permeability was not measured in this study.

Independent of the emergence of IBS following a GI infection, sickness behaviors and depressive like behaviors are notable consequences of peripheral infections (Dantzer et al., 2008). Several enteric pathogens have been shown to have a detrimental effect on the intestinal barrier (Paesold et al., 2002; Berkes et al., 2003; Flynn and Buret, 2008) which can occur via paracellular (Ferrier et al., 2003; Wu et al., 2011) or transcellular routes (Kalischuk et al., 2009). It is well established that GI infection produces elevated anxiety-like behavior in mice (Lyte et al., 2006). Antibiotics can also alter the diversity of the composition of the gut microbiota (Dethlefsen and Relman, 2011; Willing et al., 2011) with potential implications for neurochemistry and behavior (Bercik and Collins, 2014). In addition, certain antibiotics may have an adverse effect on the intestinal barrier. For example, rats administered oral clindamycin for four days, followed by oral infection with *Salmonella enteritidis* showed increased intestinal permeability as measured by 24 h urinary CrEDTA compared to the uninfected control group (van Ampting et al., 2010). Similarly, inflammatory bowel disease (IBD), a GI disease characterized by overt inflammation, is also associated with intestinal barrier dysfunction (Laukoetter et al., 2008; Marchiando et al., 2010; Antoni et al., 2014) increased intestinal permeability (Gerova et al., 2011) immune dysregulation and an altered gut microbiota (Sartor and Mazmanian, 2012). IBD is also associated with a higher prevalence of anxiety and depressive disorders (Walker et al., 2008). Moreover, stress can adversely affect the course of IBD (Mittermaier et al., 2004; Mawdsley and Rampton, 2005). Both Crohn's disease and ulcerative colitis exhibit alterations in the expression of the tight junction proteins, claudin and occludin (Heller et al., 2005; Zeissig et al., 2007). Interestingly, recent preclinical evidence suggests that chronic intestinal inflammation alters hippocampal neurogenesis (Zonis et al., 2015) which itself in influenced by the gut microbiota (Ogbonnaya et al., 2015).

## Candidate pathways to barrier dysfunction

### Serotonin

Serotonin is a critical signaling molecule in the brain-gut-microbiota axis (O'Mahony et al., 2015) and is involved in a wide range of physiological functions. In the GI tract it plays an important role in secretion, sensing and signaling (Mawe and Hoffman, 2013). The largest reserve of 5-HT is located in enterochromaffin cells (Berger et al., 2009). Emerging evidence also suggests that the serotonergic system may be under the influence of gut microbiota, especially, but not limited to, periods prior to the emergence of a stable adult-like gut microbiota (Desbonnet et al., 2008; El Aidy et al., 2012; Clarke et al., 2013). Mucosal 5-HT has been demonstrated to play a direct role in the regulation of intestinal permeability. 5-hydroxytryptophan (5-HTP), a precursor of 5HT, significantly decreased intestinal permeability in healthy control subjects and this was associated with a redistribution of ZO-1. Whilst in IBS patients 5-HTP resulted in a further decrease in occludin expression (Keszthelyi et al., 2014). The gut microbiota itself is also an important, but frequently overlooked, regulator of 5-HT synthesis and secretion. For example, colonic tryptophan hydroxylase 1 (Tph1) mRNA and protein were increased in humanized GF and conventionally raised mice. Bacterial metabolites have also been demonstrated to influence Tph1 transcription in a human enterochromaffin cell model (Reigstad et al., 2015). Others have demonstrated that distinct microbial metabolites produced by spore forming bacteria increase colonic and blood 5-HT in chromaffin cell cultures (Yano et al., 2015).

### Toll-like receptors

Toll-like receptors (TLRs) are evolutionarily conserved type I transmembrane proteins that function as pattern recognition receptors (PRRs) and recognize microbial components (Pålsson-McDermott and O'Neill, 2007; Mogensen, 2009; McCusker and Kelley, 2013). TLRs recognize microbe-associated molecular patterns (MAMPs) which are shared by many microorganisms. TLRs are expressed by a number of immune cells, including dendritic cells (DCs), macrophages, neutrophils, T cells, and B cells but are also found on non-immune cells, such as epithelial and endothelial cells (Hopkins and Sriskandan, 2005). Activation of TLRs initiates signal transduction pathways and triggers the expression of genes that control innate immune responses and further guide development of antigen-specific acquired immunity (Akira and Takeda, 2004). Thus, TLRs might serve as a molecular channel between microbiota alterations and immune homeostasis (Rogier et al., 2015). As well as playing a role in maintaining intestinal barrier function (Cario et al., 2004; Rakoff-Nahoum et al., 2004), TLRs also promote epithelial cell proliferation, secretion of IgA into the gut lumen and expression of antimicrobial peptides (Abreu, 2010). Dysregulation of these processes, or excessive TLR activation, can result in chronic inflammatory and over-exuberant repair responses. Recent evidence suggests that the TLR3 synthetic agonist, Poly(I:C) not only decreases epithelial resistance in the small intestine but also promoted thinning of the mucosal layer (Moyano-Porcile et al., 2015).

### Short chain fatty acids

The microbiota produces several bioactive metabolic products, including polysaccharides, lycosylceramides, nucleic acids, structural proteins, and SCFAs (Olle, 2013; Russell et al., 2013). SCFAs (butyrate, acetate, and propionate) are neurohormonal signaling molecules produced by certain classes of bacteria such as *Bacteroides, Bifidobacterium, Propionibacterium, Eubacterium, Lactobacillus, Clostridium, Roseburia*, and *Prevotella* (Macfarlane and Macfarlane, 2012). SCFAs are transported by monocarboxylate transporters, which notably are expressed at the BBB (Steele, 1986; Vijay and Morris, 2014). Indeed a recent preclinical imaging study demonstrated that microbiota-derived acetate can cross the BBB where it can subsequently alter hypothalamic gene expression (Frost et al., 2014). SCFAs are also pivotal in the maintenance of the intestinal barrier (Peng et al., 2007), with physiological concentrations having demonstrable effects on intestinal barrier function (Suzuki et al., 2008). Butyrate has been demonstrated to influence expression of tight junction proteins including claudin-2, occludin, cingulin, and zonula occludens proteins (ZO-1, ZO-2; Plöger et al., 2012). Butyrate has also been shown to facilitate the association between transcription factors and the claudin-1 promoter (Wang et al., 2012a), increase AMP-activated protein kinase (AMPK) activity (Peng et al., 2009) and to reduce bacterial translocation (Lewis et al., 2010). Interestingly, given the importance of butyrate in the maintenance of the intestinal barrier, IBS has been associated with a reduction in butyrate producing gut micro-organisms (Pozuelo et al., 2015). It has, however, proven difficult thus far to demarcate the CNS consequences of SCFA-mediated effects on intestinal barrier function from a direct action in the brain. It is also notable that there is still considerable debate surrounding the ability of physiological levels of SCFAs to impact substantially on relevant behaviors via central mechanisms, albeit that higher doses do have clear behavioral consequences (MacFabe et al., 2007; Macfabe, 2012).

Other mechanisms by which the microbiota may signal to the underlying mucosa, or mucosal immune system, include delivery to an underlying subset of dendritic cells via small intestine goblet cells (Artis, 2008; McDole et al., 2012). It has also been postulated that bacterial components can cross the intestinal barrier in small lipoprotein vesicles called exosomes which contain protein, nucleic acids, sugars and lipids. These exosomes can then transfer from dendritic cells to T cells in the draining lymph nodes and enter the circulation (Smythies and Smythies, 2014b). Consequently, T cells may receive epigenetic material from gut bacteria, either by direct endocytosis, or via afferent exosomes (Smythies and Smythies, 2014a). More recently, identification of “neuropods” as a pathway by which bacteria can communicate via intestinal enterochromaffin cells to the nervous system provides further insight into pathways responsible for gut to brain communication (Bohórquez et al., 2015).

### Restoration and maintenance of a healthy intestinal barrier

Diet, composition of the gut microbiota and health are intrinsically linked (Daniel et al., 2014; David et al., 2014). Diets consisting of fast food and processed food have been associated with increased intestinal permeability and depressive symptoms (Sánchez-Villegas et al., 2012). Conversely, diets rich in vegetables, fruit and fish are associated with lower depressive symptoms (Akbaraly et al., 2009; Jacka et al., 2010, 2011, 2014; Ruusunen et al., 2014). Both preclinical and clinical studies show that dietary components can alter intestinal permeability (Cani et al., 2008; Ulluwishewa et al., 2011; Stenman et al., 2012; Teixeira et al., 2012; Hamilton et al., 2015). In particular, high fat diets are associated with a greater translocation of LPS across the gut wall (Moreira et al., 2012). Moreover, meals rich in fiber and fruit have been demonstrated to reduce high fat/high carbohydrate meal-induced increases in plasma LPS levels, the inflammatory response and the expression of TLR2 and TLR4 (Ghanim et al., 2009). High fat diets can also influence the gut microbiota and have been demonstrated to increase the *Firmicutes: Bacteriodetes* ratio and induce the growth of *Enterobacteriaceae* (Kim et al., 2012). This effect was accompanied by increased plasma and fecal endotoxin levels and reduced expression of the tight junction proteins claudin-1 and occludin in the colon (Kim et al., 2012). High fat diet-induced increases in LPS translocation and barrier function are also sensitive to restoration by organisms such as *Akkermansia muciniphila* (Everard et al., 2013).

### Pre- and pro-biotics

A probiotic is defined as a live bacteria which when administered in adequate amounts confers a health benefit on the host (WHO, 2001; Petschow et al., 2013). Although, previous probiotic health claims have been exaggerated (Shanahan, 2002; Sanders, 2003; Hoffmann et al., 2013) it is clear that certain strains demonstrate beneficial effects on the intestinal barrier across the lifespan via a number of mechanisms and mediators (Eutamene and Bueno, 2007; Mennigen and Bruewer, 2009). The term “*psychobiotics*” has been recently conceived to encompass the sub-types of probiotics that may be capable of modulating the brain-gut-microbiota axis to have a beneficial effect on mood, anxiety and cognition (Dinan et al., 2013). However, despite Logan and Katzman's initial proposal of an augmenting role of probiotics in depression (Logan and Katzman, 2005), there are currently no published studies of the clinical use of probiotics in depressed patients. One double-blind, placebo-controlled trial of probiotic supplementation conducted in patients diagnosed with schizophrenia, failed to show superiority over placebo supplementation (Dickerson et al., 2014). Several studies have been conducted in the healthy population (see Table 3). In the context of primarily non-psychiatric disorders (see also Table 3), *B. lactis* augmented formula, fed to preterm infants, resulted in decreased intestinal permeability as measured by the lactulose/mannitol ratio at two, seven and 30 days post birth (Stratiki et al., 2007). In a double-blinded, placebo-controlled, cross-over study *L. rhamnosus* 19070-2 and *L. reuteri* DSM 12246 were administered for six weeks to 41 children with moderate and severe atopic dermatitis. This probiotic combination decreased associated GI symptoms and influenced small intestinal permeability as measured by the lactulose–mannitol test (Rosenfeldt et al., 2004). In healthy adults *L. plantarum* WCFS1 administered to the duodenum via feeding catheter, increased ZO-1 and occludin compared to placebo. In the *in vitro* arm of the same study *L. plantarum* induced translocation of ZO-1 to the tight junction region in human epithelium, though the authors point out that the effects on occludin were minor compared with those seen *in vivo*. Furthermore, *L. plantarum* was shown to activate TLR2 signaling (Karczewski et al., 2010). In a randomized, double-blinded, placebo controlled trial, a 14 week course of probiotic supplementation in athletes resulted in a decrease in fecal zonulin (see Table 1) in the probiotic group compared to the control group after a period of intense exercise measured at baseline and at 14 weeks (Lamprecht et al., 2012).

Some of the strongest evidence for the clinical role of probiotics comes from studies in patients with the brain-gut-axis disorder, IBS (Whelan and Quigley, 2013; Orel and Kamhi Trop, 2014). A number of probiotics and commensal organisms, primarily lactic acid bacteria, have been shown to ameliorate certain IBS symptoms (Hoveyda et al., 2009; Clarke et al., 2012; Ortiz-Lucas et al., 2013; Yoon et al., 2014; Didari et al., 2015). Some of these beneficial effects may, at least, relate to the anti-inflammatory effects of particular organisms (O'Mahony et al., 2005). Moreover, probiotics in accordance with preclinical evidence (see Table 2) can improve intestinal barrier function under pathological conditions in human populations. In a randomized single blind placebo controlled study a fermented milk drink containing *Streptococcus thermophilus, L. bulgaricus, L. acidophilus*, and *B. longum* decreased small intestinal permeability, though colonic permeability was unaltered (Zeng et al., 2008).

**Table 2:** **Preclinical studies of probiotics and intestinal barrier**.

**Probiotic**	**Effects**	**References**
VSL#3	Normalization of colonic physiologic function and barrier integrity; reduction in mucosal secretion of TNFα and IFNγ and an improvement in histologic disease	Corridoni et al., 2012
	Decreased ileal paracellular permeability, decrease claudin-2 and increase occludin in a mouse model of ileitis	
VSL#3 (protein soluble factor)	Enhanced barrier function and resistance to *Salmonella* invasion	Madsen et al., 2001
VSL#3	Prevented the increase in epithelial permeability in DSS-induced acute colitis and prevented the decrease in expression and redistribution of occludin, zonula occludens-1, and claudin-1, -3, -4, and -5	Mennigen et al., 2009
VSL#3	VSL#3 attenuated intestinal barrier damage and reduced bacterial translocation in an LPS induced mouse model of sepsis	Ewaschuk et al., 2007
*Lactobacillus rhamnosus* and *L. acidophilus*	Attenuated the damage caused by *Shigella dysenteriae*	Moorthy et al., 2009
*Lactobacillus plantarum*	Prevented the rearrangement of claudin-1, occludin, JAM-1 and ZO-1 proteins induced by *Escherichia coli*	Qin et al., 2009
*Lactobacillus reuteri*	Reduced levels of colonic mucosal adherent and translocated bacteria and attenuated the development of the colitis in interleukin IL-10 gene deficient mice	Madsen et al., 1999
*Lactobacillus rhamnosus GG* culture supernatant (LGG-s)	Pretreatment significantly inhibited alcohol-induced intestinal permeability defects, endotoxemia and subsequent liver injury	Wang et al., 2012b
*Lactobacillus rhamnosus OLL2838* (live and heat-killed)	Protected against the increase in mucosal permeability associated with DSS-induced colitis	Miyauchi et al., 2009
	Increased expression of ZO-1 and myosin light-chain kinase in intestinal epithelial cells isolated from mice of the heat-killed OLL2838 group	
*Lactobacillus casei* DN-114 001 (lysate—Lc)	Increased the numbers of CD4(+)FoxP3(+) Tregs in mesenteric lymph nodes, decreased the production of TNFα and IFNγ, and anti-inflammatory IL-10 in Peyer's patches and the large intestine; changed the gut microbiota composition in DSS colitis	Zakostelska et al., 2011
	Lc also resulted in a significant protection against increased intestinal permeability and barrier dysfunction	
	Lc treatment prevented LPS-induced TNFα expression in RAW 264.7 cell line by down-regulating the NF-kB signaling pathway	
*Lactobacillus brevis SBC8803* polyphosphate (poly P)	Suppressed the oxidant-induced increase in intestinal permeability in the mouse small intestine	Segawa et al., 2011
	Daily intrarectal administration of poly P improved the inflammatory profile and survival rate when administered to DSS mice	
*Lactobacillus rhamnosus GG*, soluble secretory proteins p40 and p75	Prevented hydrogen peroxide induced redistribution of occludin, ZO-1, E-cadherin, and beta-catenin from the intercellular junctions	Seth et al., 2008
*Lactobacillus plantarum DSM 2648*	Attenuated the negative effect of enteropathic *Escherichia coli* (EPEC) O127:H6 (E2348/69) on transepithelial electrical resistance and adherence to intestinal cells	Anderson et al., 2010a
*Lactobacillus* plantarum *MB452*	19 TJ related genes had altered expression levels including those encoding occludin and its associated plaque proteins that anchor it to the cytoskeleton	Anderson et al., 2010b
	*L. plantarum* MB452 altered tubulin and proteasome gene expression levels	
*Lactobacillus acidophilus* protects TJ from aspirin-induced damage in HT-29 cells	Protects TJ from aspirin-induced damage in HT-29 cells	Montalto et al., 2004
*Lactobacillus rhamnosus GG (LGG)* - modified lipoteichoic acid (LTA)	Correlated with a significant down-regulation of TRL2 expression and downstream proinflammatory cytokine expression in DSS mouse model	Claes et al., 2010
*Lactobacillus plantarum 299v*	Administration for one week abolished *E. coli*-induced increase in permeability	Mangell et al., 2002
*Lactobacillus helveticus and Lactobacillus rhamnosus*	Administration one week prior to, and concurrently with, *Citrobacter rodentium* attenuated *C. rodentium*-induced barrier dysfunction, epithelial hyperplasia, and binding of the pathogen to host colonocytes	Rodrigues et al., 2012
*Lactobacillus rhamnosus LOCK0900, L. rhamnosus LOCK0908* and *L. casei LOCK0919*	Colonization of GF mice enhanced the integrity of gut mucosa and ameliorated allergic sensitization	Kozakova et al., 2015
*Lactobacillus fermentum AGR1487* - cell surface structures and supernatant	Live and dead AGR1487 decreased TEER across Caco-2 cells Only live AGR1487 increased the rate of passage of mannitol	Sengupta et al., 2015
*Lactobacillus rhamnosus GG* (live or heat-killed)	Enteral administration accelerated intestinal barrier maturation and induced claudin 3	Patel et al., 2012
*Lactobacillus helveticus R0052* and *Bifidobacterium longum R0175*	Reversed the deficits in intestinal permeability and depressive like behaviors post MI	Arseneault-Breard et al., 2012
*Bifidobacterium lactis CNCM I-2494*	Prevented the increase in intestinal permeability induced by PRS and restored occludin and JAM-A expressions to control levels	Agostini et al., 2012
*Bifidobacteria infantis*	In T84 cells increased TEER, decreased claudin-2, and increased ZO-1 and occludin expression, associated with enhanced levels of phospho-ERK and decreased levels of phospho-p38	Ewaschuk et al., 2008
	Prevented TNFα and IFNγ induced decrease in TEER and rearrangement of TJ proteins	
	Oral administration acutely reduced colonic permeability in mice whereas long-term BiCM treatment in IL-10-deficient mice attenuated inflammation, normalized colonic permeability and decreased colonic and splenic IFN-gamma secretion	
*Bacteroides thetaiotaomicron*	Modulated the expression of genes involved in several important intestinal functions, including nutrient absorption, mucosal barrier fortification, xenobiotic metabolism, angiogenesis, and postnatal intestinal maturation	Hooper et al., 2001
*Escherichia coli Nissle 1917*	Altered the expression and distribution of ZO-2 protein	Zyrek et al., 2007
*Escherichia coli Nissle 1917*	Colonization of GF mice resulted in an up-regulation of ZO-1 in intestinal epithelial cells at both mRNA and protein levels	Ukena et al., 2007

*TJ, tight junction; TEER, transepithelial electrical resistance; DSS, dextran sodium sulfate; PRS, partial restraint stress*.

**Table 3:** **Probiotics and clinical stress studies**.

**Probiotic**	**Duration**	***N***	**Subjects**	**Design**	**Clinical measures**	**Biological measures**	**Results**	**References**
*Lactobacillus casei Shirota* (milk drink)	21 days	124	Healthy (average age 61.8 years)	Randomized double blind placebo controlled	Mood: profile of Mood States (POMS), at baseline, 10 days and 20 days Cognition: Episodic memory Semantic memory Verbal fluency	N/a	No general effect on mood of taking the probiotic Small improvement in mood when *post-hoc* analysis of the lowest tertile mood scores were considered Decreased performance on semantic memory	Benton et al., 2007
*L. helveticus R0052 and B. Longum R0175*	30 days	30	Healthy	Double blind placebo controlled	Hopkins Symptoms Checklist (HSCL-90) Hospital Anxiety and Depression Scale (HADS) Perceived Stress Scale (PSS) Coping Checklist (CCL)	24 h Urinary free cortisol (UFC)	Reduced global severity index, somatisation, depression and anger–hostility scores in the HSCL-90 Reduced global and anxiety scores in the HADS Improved problem solving in the CCL Decrease in UFC	Messaoudi et al., 2011
*Lactobacillus casei Shirota*	60 days	35	Chronic Fatigue Syndrome	Randomized double blind placebo controlled	Beck Anxiety and Depression Inventories	Fecal	Decrease in Anxiety symptoms Increase in *Lactobacillus* and *Bifidobacteria* in Fecal samples	Rao et al., 2009
*Clostridium Butyricum*	14 days (twice daily)	30 20 Healthy controls	Pre-op laryngectomy	Randomized, placebo controlled	Hamilton Anxiety Scale (HAMA)	Serum CRF Heart rate (HR)	Reduced anxiety levels from 19.8 to 10.2 in the HAMA Attenuated the increase in CRF and HR pre op	Yang et al., 2014
Bifidobacterium animalis, Streptococcus thermophiles, Lactobacillus bulgaricus, and Lactobacilluslactis (fermented milk)	28 days	12	Healthy Females	Randomized placebo controlled parallel-arm design		fMRI: emotional faces attention task	Reduced task related response of a distributed functional network containing affective, viscerosensory and somatosensory cortices independent of self-reported GI symptoms	Tillisch et al., 2013
*Bifidobacterium bifidum W23, Bifidobacterium lactis W52, Lactobacillus acidophilus W37, Lactobacillus brevis W63, Lactobacillus casei W56, Lactobacillus salivarius W24, and Lactococcus lactis (W19 and W58)*	28 days	40	Healthy	Triple-blind, placebo-controlled, randomized	Leiden index of depression sensitivity scale	N/a	Reduction in rumination and aggressive thoughts, subscales on the Leiden index of depression sensitivity scale	Steenbergen et al., 2015

A prebiotic effect is defined as the selective stimulation of growth and/or activity(ies) of one or a limited number of microbial genus(era)/species in the gut microbiota that confer(s) health benefits to the host (Gibson, 2004; Roberfroid et al., 2010). Preclinical and clinical studies demonstrate that certain prebiotics alter the gut microbiota, can reduce low grade inflammation and improve metabolic function (Everard et al., 2011; Greiner and Bäckhed, 2011; da Silva et al., 2013; Dewulf et al., 2013; Bindels et al., 2015). Evidence also suggests that prebiotic galacto-oligosaccharides (GOS) can improve intestinal barrier function in rats (Zhong et al., 2009). Moreover, mice treated with prebiotics exhibit improvements in intestinal permeability, tight junction integrity, decreased plasma LPS and cytokine levels in addition to decreased hepatic expression of inflammatory and oxidative stress markers (Cani et al., 2009). Prebiotics have also been shown to influence brain neurochemistry and behavior. GOS, for example, increased hippocampal neurotrophin levels and the expression N-methyl-d-aspartate receptor subunits in the rat frontal cortex (Savignac et al., 2013). Behaviorally, a combination of GOS and polydextrose attenuated anxiety-like behavior in rats and induced alterations in the gut microbiota composition (Mika et al., 2014). In the context of IBS, GOS also appeared to influence the gut microbiota and improved anxiety scores (Silk et al., 2009). Furthermore, in healthy adults, administration of GOS significantly decreased the cortisol awakening response and decreased attentional vigilance to negative vs. positive information in a dot-probe task compared to placebo (Schmidt et al., 2015).

### Conclusion and future perspectives

**Figure 2:**
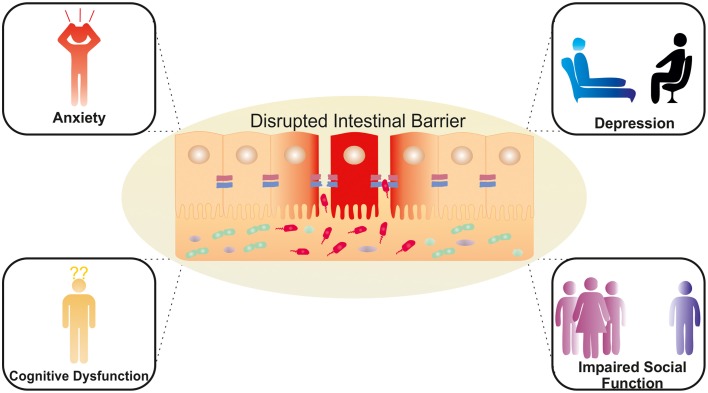
**Potential neuropsychiatric consequences of a dysregulated intestinal barrier**. Activation of brain-gut-microbiota Axis signaling pathways via a compromised intestinal barrier with potential effects on mood, anxiety, cognition and social interaction.

The area of microbiome research needs to move beyond purely compositional assessments to understanding the potential mechanisms by which dysbiosis contributes to the pathophysiology of disease. Preclinical research points to a role of the gut microbiome in brain function and behavior with a number of potential pathways being investigated. The possibility that these effects might be mediated by alterations in intestinal permeability is supported by converging lines of evidence. This includes evidence linking stress to both compromised barrier function and microbiota disruption with the ensuing systemic inflammation mediating the impact on the expression of neuropsychiatric symptoms (see Figure 2). This hypothesis needs to be elaborated in preclinical and clinical studies to establish the relevance of this proposed mechanism. Such studies will identify whether targeting the epithelial barrier is a viable strategy in managing stress-related disorders, given the established role for the gut microbiota in maintaining barrier function. Indeed, there is growing evidence that certain probiotic strains as well as prebiotics can benefit barrier function, albeit largely in healthy controls or non-psychiatric populations including stress-related GI disorders. However, the success of this strategy will be contingent on identifying sub groups of patients with a compromised epithelial barrier who may be more likely to respond to probiotics with barrier enhancing effects. Alternative approaches such as psychobiotics may target other brain-gut axis pathways independent of any effect on the intestinal barrier. Intervention studies utilizing therapeutic modulation of the gut microbiota or its metabolites to restore “normal” intestinal permeability will may be of benefit in stress-related disorders. As we discussed throughout this review, the impact of a variety of factors implicated in barrier function, such as SCFAs, could also produce their effects at higher centers independently of a compromised intestinal barrier. One clear example of a distinct alternative signaling mechanism between the gut microbiota and the CNS is via the vagus nerve (Bravo et al., 2011). In addition to the disorders mentioned in this review, preclinical evidence suggests that the gut microbiota modulates behaviors associated with neurodevelopmental disorders such as autism spectrum disorder and schizophrenia (Hsiao et al., 2013; Desbonnet et al., 2014; Mayer et al., 2014b). However, the clinical implications and the role of the intestinal barrier have yet to be fully established (Julio-Pieper et al., 2014; Mayer et al., 2015). We acknowledge that the associations between a “leaky gut” and stress-related psychiatric disorders are not without controversy and the narrative surrounding the impact of the gut microbiome on the CNS requires further development, both mechanistically and with respect to translation from promising preclinical findings to solid clinical relevance. Integrating these important observations within the theoretical framework of this review may provide the impetus to clearly establish the clinical significance of these intriguing concepts and the potential inherent in this field supports further investment and concerted efforts in this area. This success of this investigation holds promise for novel treatment options and preventative plans to support good mental health and to counteract the negative consequences of excessive stress loads.

### Conflict of interest statement

The Microbiome Institute has conducted studies in collaboration with several companies including GSK, Pfizer, Wyeth and Mead Johnson. The authors declare that the research was conducted in the absence of any commercial or financial relationships that could be construed as a potential conflict of interest.
